# Hamster Polyomavirus Research: Past, Present, and Future §

**DOI:** 10.3390/v13050907

**Published:** 2021-05-13

**Authors:** Burkhard Jandrig, Hans Krause, Wolfgang Zimmermann, Emilija Vasiliunaite, Alma Gedvilaite, Rainer G. Ulrich

**Affiliations:** 1 Department of Urology, University Medical Center Magdeburg, Leipziger Str. 44, 39120 Magdeburg, Germany; 2 Charité—Universitätsmedizin Berlin, Urologische Klinik, Charitéplatz 1, 10117 Berlin, Germany; hans.krause@charite.de; 3 LGC Genomics, BU Sequencing, Ostendstr. 25, 12459 Berlin, Germany; mwzimmermann77@alice-dsl.net; 4 Institute of Biotechnology, Life Sciences Center, Vilnius University, Saulėtekio al. 7, LT-10257 Vilnius, Lithuania; emilija.vasiliunaite@gmc.vu.lt (E.V.); alma.gedvilaite@bti.vu.lt (A.G.); 5 Institute of Novel and Emerging Infectious Diseases, Friedrich-Loeffler-Institut, Federal Research Institute for Animal Health, Südufer 10, 17493 Greifswald-Insel Riems, Germany; rainer.ulrich@fli.de; 6 German Center for Infection Research (DZIF), Partner Site Hamburg-Lübeck-Borstel-Insel Riems, Germany

**Keywords:** rodent polyomaviruses, virus discovery, Syrian hamster, genome organization, middle T antigen, tumor model, major capsid protein, virus-like particles

## Abstract

Hamster polyomavirus (Mesocricetus auratus polyomavirus 1, HaPyV) was discovered as one of the first rodent polyomaviruses at the end of the 1960s in a colony of Syrian hamsters (*Mesocricetus auratus*) affected by skin tumors. Natural HaPyV infections have been recorded in Syrian hamster colonies due to the occurrence of skin tumors and lymphomas. HaPyV infections of Syrian hamsters represent an important and pioneering tumor model. Experimental infections of Syrian hamsters of different colonies are still serving as model systems (e.g., mesothelioma). The observed phylogenetic relationship of HaPyV to murine polyomaviruses within the genus *Alphapolyomavirus,* and the exclusive detection of other cricetid polyomaviruses, i.e., common vole (Microtus arvalis polyomavirus 1) and bank vole (Myodes glareolus polyomavirus 1) polyomaviruses, in the genus *Betapolyomavirus*, must be considered with caution, as knowledge of rodent-associated polyomaviruses is still limited. The genome of HaPyV shows the typical organization of polyomaviruses with an early and a late transcriptional region. The early region encodes three tumor (T) antigens including a middle T antigen; the late region encodes three capsid proteins. The major capsid protein VP1 of HaPyV was established as a carrier for the generation of autologous, chimeric, and mosaic virus-like particles (VLPs) with a broad range of applications, e.g., for the production of epitope-specific antibodies. Autologous VLPs have been applied for entry and maturation studies of dendritic cells. The generation of chimeric and mosaic VLPs indicated the high flexibility of the VP1 carrier protein for the insertion of foreign sequences. The generation of pseudotype VLPs of original VP1 and VP2–foreign protein fusion can further enhance the applicability of this system. Future investigations should evaluate the evolutionary origin of HaPyV, monitor its occurrence in wildlife and Syrian hamster breeding, and prove its value for the generation of potential vaccine candidates and as a gene therapy vehicle.

## 1. Introduction

Right from the start of developing the concepts of modern molecular biology and tumor biology, viruses such as the simian virus 40 (SV40; Macaca mulatta polyomavirus 1) and the murine polyomavirus (Mus musculus polyomavirus 1) have served as models for a fundamental understanding of eukaryotic gene regulation and gene expression as well as tumor induction and progression [1].

Polyomaviruses are nonenveloped circular double-stranded DNA viruses that infect a variety of vertebrate host species. In humans, mainly four polyomaviruses have been strongly associated with clinical disease. The Human polyomavirus 1 (HPyV-1; BK polyomavirus, BKPyV), Human polyomavirus 2 (HPyV-2, John Cunningham polyomavirus, JCPyV), Human polyomavirus 5 (Merkel cell polyomavirus, MCPyV), and Human polyomavirus 8 (Trichodysplasia spinulosa-associated polyomavirus, TSPyV) can cause severe diseases especially in immunosuppressed individuals. HPyV-1 is associated with nephropathy, a leading cause of kidney transplant failure, HPyV-2 can cause the demyelinating disease progressive multifocal leukoencephalopathy (PML), HPyV-5 is able to integrate into the cellular genome to cause a highly aggressive skin cancer (Merkel cell carcinoma), and HPyV-8 is an etiological factor for the dermatological disease Trichodysplasia spinulosa (TS). However, the mechanisms by which these viruses give rise to the relevant diseases are not well understood.

This review aims to describe the discovery and characterization of the hamster polyomavirus, its implication in the development of cancer, pathological features, and the application of its capsid-derived recombinant proteins for the generation of virus-like particles (VLPs). A timeline of objectives of hamster polyomavirus studies, leading researchers, and publication numbers are shown in Figure S1.

## 2. Hamster Polyomavirus—Its Discovery and Initial Characterization

Polyomaviruses have been identified in numerous mammalian species including members of different families of the order Rodentia [2]. Modern approaches such as generic polymerase chain reaction (PCR), rolling circle amplification, and high-throughput sequencing resulted in a rapid increase in the number of described rodent polyomaviruses, although not all of them are completely sequenced yet (see Table 1).

However, the knowledge of rodent polyomaviruses is still very scarce. This is illustrated when comparing the number of known polyomaviruses in humans (n = 14) with those in more than 2000 rodent species (n = 34, see Table 1). Whereas three distinct polyomaviruses have been described in a very few rodent species, there is still only one complete virus genome known in hamsters.

A hamster polyomavirus (HaPyV; Mesocricetus auratus polyomavirus 1; previously Hamster papovavirus, HaPV) was first isolated from skin epitheliomas arising in the Syrian hamster colony in Berlin-Buch, Germany and described by Graffi et al. [13] (a screenshot of the paper is shown in Figure S2). The tumors appeared spontaneously in young animals of about 3 months to more than one year of age. Polyomavirus infections in hamsters were also reported from the United States (Newfield, NJ [16]; Columbia, MO [17]; New Haven, CT [18]) and Europe (Bristol, GB [19]; Salamanca, Spain [20]). In general, HaPyV infections are rarely reported. Screening for polyomaviruses in laboratory hamsters is not officially required by the Federation of European Laboratory Animal Science Associations [21] and not rigorously demanded by local supervisory authorities.

Virus particles extracted from skin epithelioma caused lymphoma and leukemia when injected into newborn hamsters from a distinct and tumor-free colony bred in Potsdam, Germany [22]. The role of HaPyV as the causative agent of transmissible lymphoma was further investigated by Barthold et al. [23]. Lymphoma developed not only in the originally infected hamsters but also in hamsters maintained in direct and indirect contact. In addition, one of the contact hamsters developed cutaneous virus-containing epitheliomas. A hallmark of HaPyV is the capacity to infect both undifferentiated keratinocytes as well as lymphocytes and thereby to generate hair follicle and lymphoid tumors.

HaPyV particles were first detected on electron micrographs of primary skin sections [13]. The particles, about 40 nm in diameter, accumulated in large amounts in skin epithelioma. These are spherical particles with the typical icosahedral structure of polyomaviruses that are characterized by a molecular weight of 27.5 × 10^6^, a sedimentation coefficient of 223 S, and a buoyant density of 1.340 g/mL [24]. The symmetry of the capsid was classified as T = 7 laevo [25]. Virus particles are assembled in the nuclei of keratinized cell layers and are abundant in the differentiated *stratum corneum* but absent in the proliferating cells of the *stratum basale* and *stratum spinosum*. In situ hybridization of whole body animal sections conducted before the appearance of epitheliomas demonstrated that thymus and spleen represent the most active virus reservoirs [26]. Accordingly, an accumulation of virus particles could be observed on electron micrographs of hamster thymus. In contrast to primary skin epitheliomas, virus particles could not be detected on electron micrographs of transplanted skin tumors. The absence of detectable viral genomes in total embryo tissues supports the model of horizontal transmission [17,19]. Although lymphomas induced by HaPyV were carefully examined for the presence of virus particles by electron microscopy, virus particles could not be found. Instead, different amounts (sometimes several thousands of copies) of extrachromosomal nonrandomly deleted free and/or integrated viral genomes accumulated in the tumors [20,26,27].

## 3. HaPyV Model Systems

The availability of experimental models is necessary in order to study the induction and growth processes of tumors and metastases. Experimental Syrian hamster models were created by the subcutaneous inoculation of virions isolated from skin tumors and HaPyV DNA purified from the virions [28]. Out of skin tumors, a spectrum of tumors was induced in a Syrian hamster colony, mainly lymphomas (>50%), but also sarcomas (8%) and mesotheliomas (2%) [29]. Mesothelioma became important because of its occurrence in humans after exposure to asbestos and also to non-asbestos-related noxa, e.g., mineral oils, metal dust, and opaque matter applications. Interestingly, the Syrian hamsters bearing primary mesotheliomas were female. This sex difference was maintained in transplantation experiments demonstrating an accelerated tumor growth in female as compared to male animals. The preference of female animals in mesothelioma induction and growth remains unclear. 

Transgenic mice have been obtained by the microinjection of HaPyV supercoiled DNA into pronuclei of fertilized eggs of Gat:NMRI mice [30]. Analysis of different tissues in three generations showed the presence of the HaPyV transgene as extrachromosomal DNA and its expression preferentially in the thymus and the spleen. At the age of 18 months, four of seven founders developed skin papillomas histologically similar to that of the random-bred hamster tumors. In these epitheliomas, high amounts of episomal viral genomes were accumulated, whereas virus particles could not been detected. Two founder mice lines have developed lymphomas containing extrachromosomal viral genomes. Lymphomas and papillomas have been observed in 5- to 9-month-old F1 animals. F2 litters are affected by severe developmental and often lethal damage e.g., almost complete thymus depletion. 

Transgenic mice in which the expression of the reporter gene lacZ is driven by the HaPyV early promoter have shown that this promoter displays a rather specific expression pattern restricted to hematopoietic organs, mainly thymus and spleen [31].

Reflection on former models could give important insights into carcinogenic processes and may open new therapeutic options.

## 4. HaPyV as Pathogen

HaPyV is transmitted horizontally via urine [32] or by biting and grooming behaviors. Infection is mainly associated with trichoepitheliomas (hair follicle tumors) and/or lymphoma or leukemia. In addition, the animals are suffering from subclinical effects, which mainly occur in older hamsters. There, the virus persists in the renal tubular epithelium. Trichotheliomas in hamsters have only been described in association with an HaPyV infection. HaPyV induces hair follicle keratinocyte proliferation, especially of the hair root epithelium. Alopecic nodules mainly develop on the head and spread to the neck, back, belly, and feet. Sometimes, multiple nodules converge to massive confluent layers [33]. Histologically, the tumors form cyst-like masses filled with cornified material, sometimes containing melanin. Virus particles are present especially in the cornified layer but are absent in the proliferating cells of the stratum basale and stratum spinosum. In some areas, scattered chronic inflammatory infiltrates of lymphocytes and macrophages could be observed [19].

Lymphomas are mainly associated with HaPyV infection of young virus-naïve hamsters. Mortality rates among these hamsters can reach 80% within 4 to 30 weeks following infection [17]. Affected sites are mesenteric lymph nodes and liver and with a lesser extent in kidneys, spleen, thymus, small intestine, or peripheral lymph nodes. Histologically, the neoplastic lymphocytes of B- or T-cell origin are large and immature. The invasion of adjacent tissue and metastasis occur often. The concurrent formation of skin tumors and lymphoma in a hamster individuum is a relatively rare event [18].

In an inbred colony of genetic audiogenic seizure Syrian golden hamsters (GASH:Sal) in Spain, a high incidence of non-Hodgkin, Burkitt-type-B-cell lymphoma was detected, resulting in mortality within 1 to 2 weeks [20]. An indirect ELISA and Western blot analysis confirmed the presence of antibodies against the VP1 capsid protein of HaPyV in the sera not only from affected and non-affected GASH:Sal hamsters but also from control hamsters from the same breeding area.

So far, there are no treatment options for hamsters with HaPyV infection. Aggressive environmental decontamination of the virus has failed in some facilities [19]. As an increased use of personal protective equipment has also not been successful, culling is mainly recommended when the HaPyV infection is widespread within a colony.

## 5. Genome Characterization of HaPyV

With the emergence of recombinant technology in the mid-1970s [34,35], opportunities arose to obtain the DNA genome of HaPyV in a form that could be more easily managed, thus permitting extensive molecular characterization. Finally, around 1978 rules were established for physical and biological safety, including the distribution of safe bacterial host and plasmid vectors [36].

In the early 1980s, full-length HaPyV was inserted into BamHI-site of the bacterial plasmid pBR322 [37,38] permitting initial restriction enzyme mapping as well as electron microscopic analyses [39]. This initial cloning provided viral DNA for extended studies such as heteroduplex analysis, protein-binding site investigations, evolutionary sequence analysis, and transformation experiments [40,41,42,43].

The most comprehensive molecular characterization of the HaPyV genome including the deposition of the almost entire nucleotide sequence (5366 base pairs, bp, GenBank X02449, later corrected with modern sequencing techniques to 5372 bp, GenBank JX036360) as well as a comparison to other polyomavirus genomes was carried out during the mid-1980s at the Institute Gustave Roussy, Villejuif, France in collaboration with the group of Jean Feunteun [44,45].

**Figure 1:**
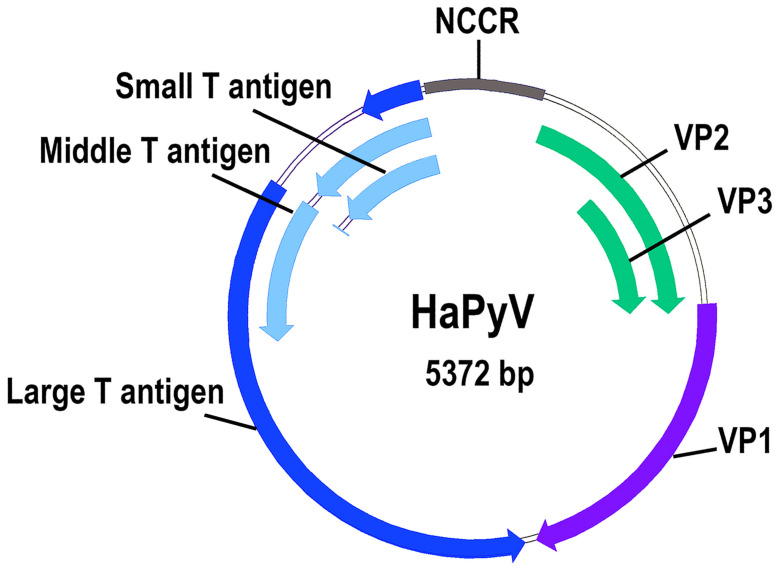
Genome organization of hamster polyomavirus. The double-stranded DNA genome of 5372 base pairs contains a noncoding control region (NCCR), an early transcriptional region encoding three T antigens and a late transcriptional region encoding capsid proteins VP1, VP2, and VP3. The coding regions are depicted as shaded green, blue, light blue, and violet arrows. As for all polyomaviruses, the proximal N-terminal region of all three T antigens is identical. The VP2 represents an N-terminally prolonged VP3; i.e., the VP3 region is identical to the C-terminal part of VP2.

With regard to viral gene expression, the early and late mRNA transcripts of HaPyV were detected on the basis of cDNA cloning and subsequent sequence determination [45] (Figure 1). 

The synthesis of the early tumor (T) antigens and late viral proteins (VP) of HaPyV were confirmed indirectly by the heterologous synthesis of protein segments of large T (LT), middle T (MT), small T (ST), VP1, and VP2/VP3 in Escherichia coli and their reactivity with sera from naturally HaPyV-infected Syrian hamsters of the Z3 strain [46]. The viral major capsid protein VP1 was additionally confirmed by its reactivity with rabbit polyclonal antibodies raised against E. coli-expressed VP1 [25].

The genome characterization of HaPyV resulted in the identification of two putative in-frame translation initiation codons of VP1-open reading frame (ORF) encoding 384 and 388 amino acid (aa) residue-long VP1 variants [25]. The heterologous expression of both VP1-ORF variants in E. coli, yeast Saccharomyces cerevisiae, and insect cells resulted in the formation of VLPs [47,48,49]. In contrast, the sequencing of purified viral VP1 (GenBank AJ006015) suggested the second AUG codon as a translation initiation site or a post-translational N-terminal cleavage of VP1 [25]. As expected, the VP2 sequence (GenBank AJ006016) represents an N-terminally prolonged VP3 sequence (GenBank AJ006017).

**Figure 2:**
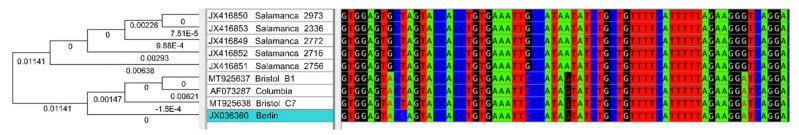
The nucleotide sequences of HaPyV strains from different Syrian hamster colonies available in GenBank were assembled and the contig formed with the phred-basecalling and the phrap-assembly tool available in the suite CodonCode Aligner v.9 (CodonCode Corp., Centerville, MA, USA). CodonCode Aligner also supported building neighbor-joining trees for the contig. The branch lengths of the neighbor-joining tree represent the distance between the samples. Part of the contig is shown on the right representing the area between nucleotides 3337 and 3404 of the Hamster polyomavirus isolate Berlin (GenBank JX036360). This area is located within the VP1 gene.

Nucleotide sequences of HaPyV deposited in GenBank were compared using the multiple sequence alignment program MUSCLE embedded in CodonCode Aligner^TM^. A phylogenetic tree built by the neighbor-joining method based on the matrix of pair-wise genetic distances between samples shows a closer relationship of the German isolate (Berlin) to the British (Bristol) or American (Columbia) sequences than to the Spanish (Salamanca) variants (see Figure 2). However, at the moment, very limited data are available in order to make statements about potential genetic relationships.

**Figure 3:**
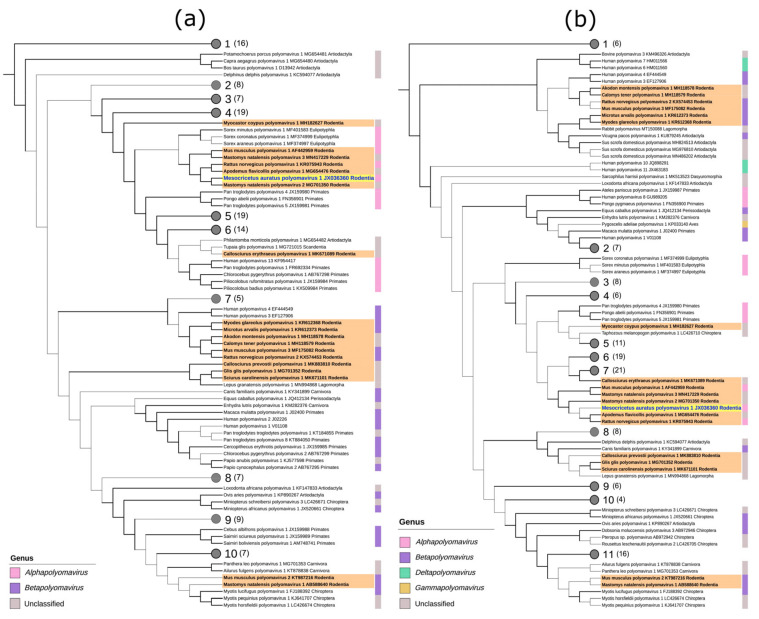
Phylogenetic trees of 174 representatives of the Polyomaviridae family obtained using LT (**a**) and VP1 (**b**) amino acid sequence alignments. Polyomaviruses are named following the recommendations of the International Committee on Taxonomy of Viruses (ICTV), accession numbers of sequences are provided. Virus genera are indicated by a colored stripe on the right side of each tree and the orders of the viral hosts are given. Rodent polyomaviruses are depicted in bold font with an orange background, hamster polyomavirus is emphasized with a bright yellow background. Trees were generated using a maximum likelihood analysis, Transfer Bootstrap Expectation support for bold black branches is >0.85. The number of leaves in condensed nodes are given in parenthesis. Detailed information on condensed nodes and tree generation and quoted references are given in Supplementary Table S1 [51,52,53,54,55,56,57,58,59].

The initial molecular characterization of the genome already suggested a similarity of HaPyV to the murine polyomavirus (MuPyV; Mus musculus polyomavirus 1), both encoding a MT antigen, but a clear divergence to the primate polyomavirus SV40 and human polyomavirus HPyV-1 (BKPyV). The initial assumption of the exclusive occurrence of the MT antigen-encoding capacity in rodent polyomaviruses [50] must be withdrawn according to new data [2]: Some of the rodent-associated polyomaviruses, such as Mastomys natalensis polyomavirus 2, Rattus norvegicus polyomavirus 1, and Apodemus flavicollis polyomavirus 1 encode a MT, but Glis glis polyomavirus 1 does not. On the opposite side, two non-rodent-associated polyomaviruses also encode an MT antigen, i.e., Tupaia glis polyomavirus 1 and Philantomba monticola polyomavirus 1. Recent phylogenetic investigations of LT and VP1 amino acid sequences confirmed the previously documented close relationship of HaPyV to MuPyV [45] but also to other murine rodent associated polyomaviruses within the genus Alphapolyomavirus (Figure 3a,b). 

Interestingly, other cricetid-associated polyomaviruses, i.e., bank vole (Myodes glareolus polyomavirus 1) and common vole (Microtus arvalis polyomavirus 1) polyomaviruses, belong to the genus Betapolyomavirus, distinct from the cricetid associated HaPyV. This current phylogenetic position of HaPyV must be considered with caution because it might be biased by the still limited knowledge of rodent-associated polyomaviruses.

## 6. Past, Present, and Future: HaPyV-Derived VLPs

**Figure 4:**
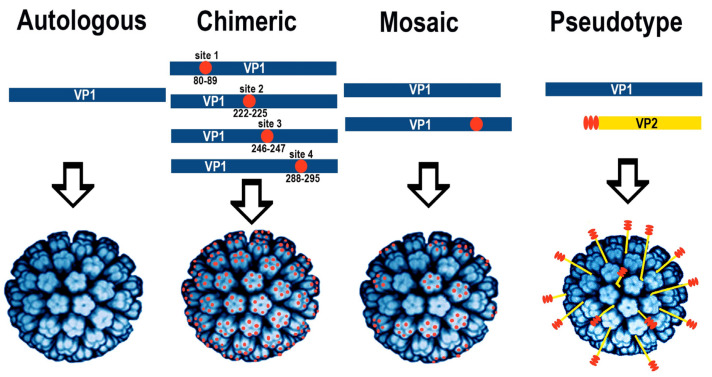
Strategies for the production of autologous, chimeric, mosaic, and pseudotype VLPs. For the generation of chimeric VLPs, four potential insertion sites were used that are located on flexible and variable regions of VP1. Mosaic VLPs are generated by the simultaneous expression of non-modified VP1 with a VP1-foreign sequence fusion protein. Pseudotype VLPs can be generated by the co-expression of VP1 and a VP2-foreign sequence fusion protein.

VLPs can be produced by heterologous synthesis of viral capsid and/or envelope proteins and represent a very useful alternative to infectious virus particles with broad applications in basic and applied research [60]. The production of VLPs follows different approaches resulting in autologous, chimeric, mosaic, and pseudotype VLPs (Figure 4).

Autologous HaPyV-VP1 derived VLPs were used to compare their uptake mechanisms by human dendritic cells (DCs) to that of VLPs of other polyomaviruses [61]. These investigations profit also from the generation of VLPs harboring an enhanced green fluorescent protein (eGFP) insertion [62]. In addition, HaPyV-VP1 derived VLPs induce maturation of human DCs and a robust T-cell response [61]. E. coli-derived HaPyV VLPs were also shown to transfer foreign plasmid DNA into mammalian cells [49].

To exploit HaPyV VP1 as a carrier for foreign epitopes in VLPs, multiple epitope mapping studies were performed to identify potential insertion sites for foreign sequences. An initial evaluation of dihydrofolate reductase (DHFR) fusion proteins with different segments of HaPyV-VP1 resulted in the identification of the C-terminus of VP1 as an immunodominant and cross-reactive region that was not only detected by sera of infected tumor bearing Z3 and tumor-free PF hamsters but also by sera raised against SV40 and HPyV-2 (JCPyV) [48]. The C-terminal region of VP1 was confirmed as immunodominant and cross-reactive by the construction of bacteriophage fr fusion proteins [63]. In addition, the formation of phage fr-capsid protein derived chimeric VLPs confirmed the flexibility of the C-terminal region of HaPyV-VP1. A PepScan analysis identified linear epitopes within VP1 that showed different reactivities with sera from tumor-bearing and papilloma-free hamsters [48].

Sequence comparisons and prediction of the structure resulted in the identification of four flexible, variable, and surface-exposed regions within VP1 of HaPyV that represent, in addition to the C-terminal region, potential insertion sites for foreign epitopes [64]. Furthermore, various truncations or amino acid exchanges within the C-terminal region indicated the robustness of the VP1 assembly capacity but also potential limitations [65].

**Figure 5:**
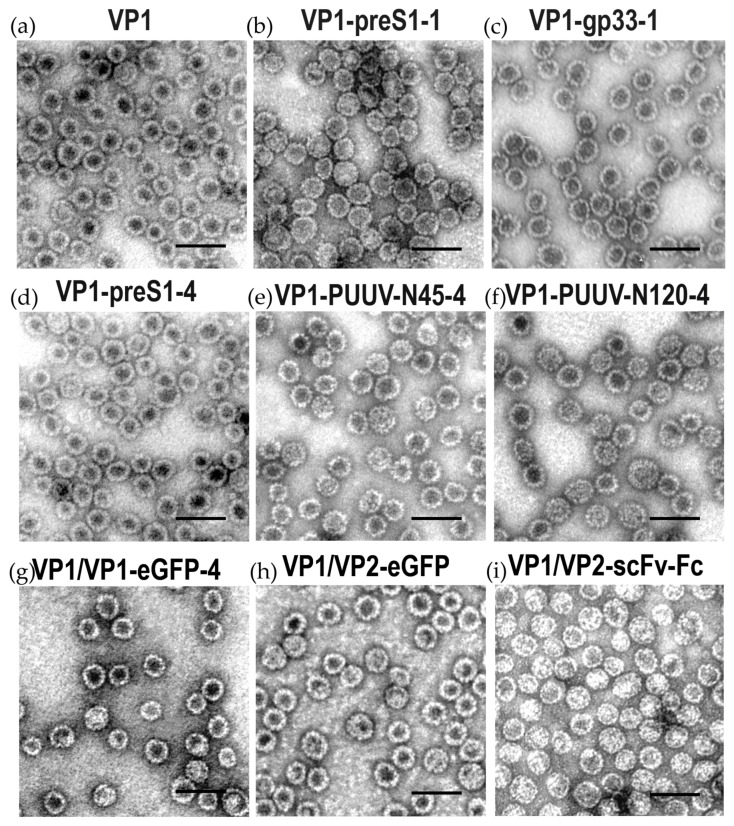
Electron-microscopical images of autologous VP1-derived virus-like particles (**a**), chimeric VP1-derived virus-like particles with hepatitis B virus preS1 peptide (**b**) or lymphocytic choriomeningitis virus gp30 peptide in insertion site #1 (**c**), HBV preS1 peptide, Puumala orthohantavirus nucleocapsid protein (N) amino acids 1–45 or 1–120 at insertion site #4 (**d**–**f**), mosaic virus-like particles with enhanced green fluorescent protein (eGFP) in site #4 (**g**) and pseudotype virus-like particles with a VP2 fusion of eGFP or single chain variable fragment with human Fc IgG domain (**h**,**i**), all produced in yeast Saccharomyces cerevisiae. The bar represents 100 nm.

The insertion of a pentapeptide of the hepatitis B virus preS1 region allowed the formation of chimeric VLPs by VP1-preS epitope fusion proteins in S. cerevisiae [64] (Table 2, Figure 5b,d). The subsequent insertion of 45 and 120 amino acid-long N-terminal segments of Puumala orthohantavirus (PUUV) nucleocapsid (N) protein suggested a high insertion capacity of the VP1 carrier protein [66] (Table 2, Figure 5e,f). 

The successful use of HaPyV-derived VLPs was continued by the insertion of various viral and non-viral epitopes and protein segments (see Table 2, Figure 5). The flexibility of this carrier system was enhanced by the insertion of flexible glycine–serine–serine–glycine (GSSG) linker sequences at the insertion sites [62,68]. Furthermore, HaPyV–VP1-derived chimeric VLPs demonstrated the potential to induce high insert-specific and long-lasting antibody responses, even without adjuvant co-application [62,68]. The strong immunogenicity was also evidenced by the generation of monoclonal antibodies to inserted Mucin 1 peptide (MUC1), PUUV N protein segment, and glycoprotein Gc segment [62]. Moreover, HaPyV VP1-derived chimeric VLPs were able to trigger the development of an effective insert-specific cytotoxic T-cell (CTL) response in vivo in mice when HaPyV-VP1 VLPs with inserted CTL epitope from surface glycoprotein of Lymphocytic choriomeningitis virus (LCMV) GP33 were used for immunization [70]. In vitro human DCs primed with HaPyV-VP1 VLPs with inserted MUC1 peptide were able to stimulate autologous peripheral blood leukocytes [61].

The high insertion capacity of the carrier was also demonstrated by simultaneous insertions of short epitopes at multiple sites of VP1 [61,62,64,68,71] (Table 2). The insertion of short peptides of interest into VP1 protein is very useful for improvement of immunogenicity of these epitopes by providing high epitope densities. Nevertheless, despite some successful insertions of long peptides into VP1 protein [62,66], the insertion capacity for long foreign peptides is limited, as it apparently affects the correct folding and assembly of the chimeric VP1 protein. The assembly of VLPs could be recovered by generating mosaic VLPs consisting of VP1+insert fusion protein and non-modified VP1 (Gedvilaite, unpublished data). The employment of VP1/VP2 pseudotype VLPs provides additional possibilities for protein engineering of the HaPyV carrier system, e.g., by increasing the insertion capacity. This approach allows the fusion of long polypeptides or protein complexes with VP2 protein, which assembles into pseudotype VLPs after co-expression with unmodified VP1 protein in the same yeast cell. HaPyV VP1/VP2-based pseudotype VLPs with inserted protein molecules displayed on the surface of VLPs were used as a carrier for different proteins such as p16^INK4A^ (156 aa), eGFP (238 aa), or even 473 aa long functionally active complex molecules such as single-chain antibody fragment fused with human antibody Fc fragment (scFv-Fc) [73,74] (Gedvilaite, unpublished data; Figure 5). The recombinant scFv-Fc antibody molecules displayed on the surface of pseudotype VLPs were stable, correctly folded and functionally active in all used applications [74,75], and demonstrated the potential of recombinant VLPs as a highly efficient carrier for functionally active complex proteins. Pseudotype VLPs represent a new type of immunogen as they were able to induce a strong immune response against the target antigen p16^INK4A^ in immunized mice [73].

## 7. Conclusions

Currently, HaPyV represents the only well-characterized polyomavirus in cricetine rodents. A large-scale polyomavirus screening in wild Syrian hamsters and other hamster species is lacking. Therefore, the phylogenetic relationship of HaPyV to murine polyomaviruses within the genus Alphapolyomavirus and the exclusive detection of other cricetid polyomaviruses, i.e., common vole and bank vole polyomaviruses, in the genus Betapolyomavirus, must be considered with caution. The re-emergence of HaPyV outbreaks in Syrian hamster breeding and experimental units may suggest the necessity to continuously monitor breeding and hygiene regimens when implementing novel animals into existing breeding colonies. Further efforts are still needed to understand the different outcomes of HaPyV infections in Syrian hamsters of different origin, because no treatment options exist, and mainly all the hamsters in a colony have to be culled. HaPyV-VP1 and -VP1/VP2-derived VLPs represent a highly flexible carrier moiety for the insertion of foreign sequences of different size and origin offering a broad range of potential applications in basic and applied research. These virus-like particles are safe, easy to produce, and can be tailored for specific delivery or epitope presentation [77]. Future investigations should evaluate the evolutionary origin of HaPyV, monitor its occurrence in wildlife and Syrian hamster breeding, and prove its value for the generation of potential vaccine candidates and as a gene therapy vehicle.

## Figures and Tables

**Table 1:** Overview about rodent-associated polyomaviruses.

Rodent Family	Subfamily	Polyomavirus Species	Polyomavirus Genus	Reference
Muridae	Murinae	Mus musculus polyomavirus 1 Mus musculus polyomavirus 2 Mus musculus polyomavirus 3	*Alphapolyomavirus* *Betapolyomavirus* *Betapolyomavirus*	[3] [4] [5]
		Mus caroli polyomavirus 1 (high-throughput sequencing-based partial sequence information)	unclassified	[6]
		Mus pahari polyomavirus 1	unclassified	[6]
		Apodemus flavicollis polyomavirus 1	*Alphapolyomavirus*	[2]
		Apodemus agrarius polyomavirus 1 (high-throughput sequencing-based partial sequence information)	unclassified	[6]
		Apodemus draco polyomavirus 1 (high-throughput sequencing-based partial sequence information)	unclassified	[6]
		Apodemus sylvaticus polyomavirus 1 (high-throughput sequencing-based partial sequence information)	unclassified	[6]
		Rattus norvegicus polyomavirus 1 Rattus norvegicus polyomavirus 2	*Alphapolyomavirus* *Betapolyomavirus*	[7] [8]
		Rattus tanezumi polyomavirus 1 (high-throughput sequencing-based partial sequence information)	unclassified	[6]
		Mastomys natalensis polyomavirus 1 Mastomys natalensis polyomavirus 2 Mastomys natalensis polyomavirus 3	*Betapolyomavirus* *Alphapolyomavirus* *Alphapolyomavirus*	[9] [2] [10]
		Niviventer confucianus polyomavirus 1 (high-throughput sequencing-based partial sequence information)	unclassified	[6]
	Gerbillinae	Meriones unguiculatus polyomavirus 1 (high-throughput sequencing-based partial sequence information)	unclassified	[6]
		Meriones meridianus polyomavirus 1 (high-throughput sequencing-based partial sequence information)	unclassified	[6]
Gliridae	Glirinae	Glis glis polyomavirus 1	*Betapolyomavirus*	[2]
Sciuridae	Sciurinae	Sciurus carolinensis polyomavirus 1	*Betapolyomavirus*	[11]
		Sciurus variegatoides polyomavirus 1 (incomplete genome sequence)	*Alphapolyomavirus*	[11]
	Callosciurinae	Callosciurus prevostii polyomavirus 1	*Betapolyomavirus*	[11]
		Callosciurus erythraeus polyomavirus 1	*Alphapolyomavirus*	[11]
Cricetidae	Arvicolinae	Myodes glareolus polyomavirus 1 (Bank vole polyomavirus)	*Betapolyomavirus*	[12]
		Microtus arvalis polyomavirus 1 (Common vole polyomavirus)	*Betapolyomavirus*	[12]
		Microtus fortis polyomavirus 1 (high-throughput sequencing-based partial sequence information)	unclassified	[6]
		Eothenomys melanogaster polyomavirus 1 (high-throughput sequencing-based partial sequence information)	unclassified	[6]
	Cricetinae	Mesocricetus auratus polyomavirus 1 (Hamster polyomavirus)	*Alphapolyomavirus*	[13]
		Cricetulus longicaudatus polyomavirus1 (high-throughput sequencing-based partial sequence information)	unclassified	[6]
	Sigmodontinae	Akodon montensis polyomavirus 1	unclassified	[14]
		Calomys tener polyomavirus 1	unclassified	[14]
Echimyidae	Echimyinae	Myocastor coypus polyomavirus 1	*Alphapolyomavirus*	[15]
Dipodidae	Dipodinae	Dipus sagitta polyomavirus 1 (high-throughput sequencing-based partial sequence information)	unclassified	[6]
	Allactaginae	Allactaga sibirica polyomavirus 1 (high-throughput sequencing-based partial sequence information)	unknown	[6]

**Table 2:** Overview about hamster polyomavirus-derived chimeric, mosaic and pseudotype virus-like particles.

Chimeric/Mosaic/Pseudotype	Insertion Sites	GSSG Linker	Foreign Insert	Formation of Chimeric/Mosaic/Pseudotype VLPs	References
Chimeric					
**VP1 with one insert**	#1 (between aa positions 80 and 89)	−L	preS1 (5 aa or 8 aa)	+	[64,67]
		−L	MUC1 (9 aa)	+	[61]
		+L	MUC1 (9 aa)	+	
		−L	CEA (9 aa)	+	[68]
		+L	CEA (9 aa)	+	
		−L	PADRE (13 aa)	+	[67]
		−L	PUUV-N (45, 80 or 120 aa)	+	[66]
		−L	hTERT (9 or 15 aa)	+/−	[69]
		−L	PUUV-Gc (99 aa)	+	[70]
		−L	LCMV gp33 (9 aa)	+	[71]
		−L	PrP (78 aa)	+	[72]
		−L	PrP (37 aa)	+	
		−L	PrP (92 aa)	+	
		−L	PrP (46 aa)	+	
	#2 (between aa positions 222 and 225)	−L	preS1 (5 aa)	+	[64]
		−L	PUUV-N (45 or 120 aa)	-	[66]
	#3 (between aa positions 243 and 247)	−L	preS1 (5 aa)	+	[64]
		−L	PUUV-N (45 or 120 aa)	-	[66]
	#4 (between aa positions 288 and 295)	−L	preS1 (5 aa)	+	[64]
		+L	preS1 (8 aa)	+	[67]
		−L	MUC1 (9 aa)	+	[61]
		+L	MUC1 (9 aa)	+	
		−L	CEA (9 aa)	+	[68]
		+L	CEA (9 aa)	+	
		+L	PADRE (13 aa)	+	[67]
		−L	PUUV-N (45 or 120 aa)	+	[66]
		−L	eGFP (238 aa)	+	[73]
**VP1 with one insert**	#4 (between aa positions 288 and 295)	−L	TRP (9 aa) +MAGE (9 aa) +HTERT (9aa)	+/−	[69]
		−L	HTERT (9 and 15 aa)	+/−	[69]
		−L	PUUV-Gc (99 aa)	+	[70]
		−L	LCMV gp33 (9 aa)	+	[71]
		+L	PrP (78 aa)	+/−	[72]
		+L	PrP (37 aa)	+/−	
		+L	PrP (92 aa)	+	
		+L	PrP (46 aa)	+	
**VP1 with two inserts**	#1+#2	−L	preS1 (5 aa)	+	[64]
	#1+#3	−L	preS1 (5 aa)	+	[64]
	#1+#4	−L	MUC1 (9 aa)	+	[62] [61]
		+L	MUC1 (9 aa)	+	
		−L	CEA (9 aa)	+	[68]
		+L	CEA (9 aa)	+	
**VP1 with three inserts**	#1+#3+#4	−L	TRP (9 aa), MAGE (9 aa). HTERT (9 aa)	+	[69]
**VP1 with four inserts**	#1+#2+#3+#4	+L	MUC1 (9 aa)	-	[61]
		+L	CEA (9 aa)	-	[68]
**Mosaic**					
**VP1 +** **VP1 with insert**	#4	−L	eGFP (238 aa)	+	Gedvilaite, unpublished data
Pseudotype					
**VP1 +** **VP2 fused with insert at its N terminus**		+L	p16^INK4A^ (156 aa)	+	[74]
		+L	eGFP (238 aa)	+	Gedvilaite, unpublished data
		+L	anti-VLY scFv-Fc (472 aa)	+	[75]
		+L	anti-HBV scFv (243 aa)	+	[76]
		+L	anti-HBV scFv-Fc (472 aa)	+	[76]
		+L	PrP (199 aa)	+	[72]
		+L	PrP (78 aa)	+	
		+L	PrP (92 aa	+	

+L, GSSG linker inserted; −L, no linker inserted; +, formation of VLPs; −, no formation of VLPs; +/−, mix of VLPs and pentamers; #1-4 different insertion sites between aa positions. Abbreviations: preS1, B-cell epitope of the surface protein of the hepatitis B virus; MUC1, CTL-epitope of human tumor-associated mucin 1; CEA, CTL-epitope of human tumor-associated carcinoembryonic antigen; PADRE, universal T cell-specific epitope; PUUV-N; nucleocapsid protein of Puumala orthohantavirus (PUUV), strain Vranica-Hällnäs; HTERT, CTL-epitope of human telomerase reverse transcriptase; PUUV-Gc, C-terminal glycoprotein segment of PUUV strain Kazan; LCMV, lymphocytic choriomeningitis virus; PrP, mouse prion protein (used peptides: 78 aa long include aa 51–128; 37 aa long include aa 128–164; 92 aa long include aa 128–219; 46 aa long include aa 174–219 and 199 aa long include aa 23–231); TRP, CTL-epitope of human tyrosinase-related protein-2; MAGE, CTL-epitope of human MAGE A family protein; eGFP, enhanced green fluorescent protein; p16^INK4A^, human protein of high diagnostic relevance considered to be a potential marker for cells transformed by high-risk human papillomavirus; anti-VLY scFv-Fc, single chain variable fragment with human Fc IgG domain specific to bacterial cytolysin vaginolysin (VLY); anti-HBV scFv, single chain variable fragment specific to hepatitis B virus (HBV) surface antigen HBsAg; anti-HBV scFv-Fc, single chain variable fragment with human Fc IgG domain specific to HBV surface antigen HBsAg; GSSG, flexible glycine-serine-serine-glycine linker.

## Data Availability

Not applicable.

## Supplementary Materials

The following are available online at https://www.mdpi.com/article/10.3390/v13050907/s1. Figure S1: Time chart of objectives of hamster polyomavirus studies, involved main persons and number of publications when searching PubMed, Figure S2: Screenshot of the title of the first paper describing a hamster polyomavirus, typical multiple skin tumors and a picture by electron microscopy [13], Table S1: Methods and sequences used for phylogenetic analyses to get phylogenetic relationships of polyomaviruses based on LTAg/VP1 amino acid sequences.
