# Supplementary material for: Hamster Polyomavirus Research: Past, Present, and Future
Source: Viruses. 2021 May 13;13(5):907. doi: 10.3390/v13050907 (PMC8153644; doi:10.3390/v13050907)
Supplement: Supplementary file 1 [file viruses-13-00907-s001.zip › Supplementary files/Jandrig_Supplementary-Figures1-2.pdf]

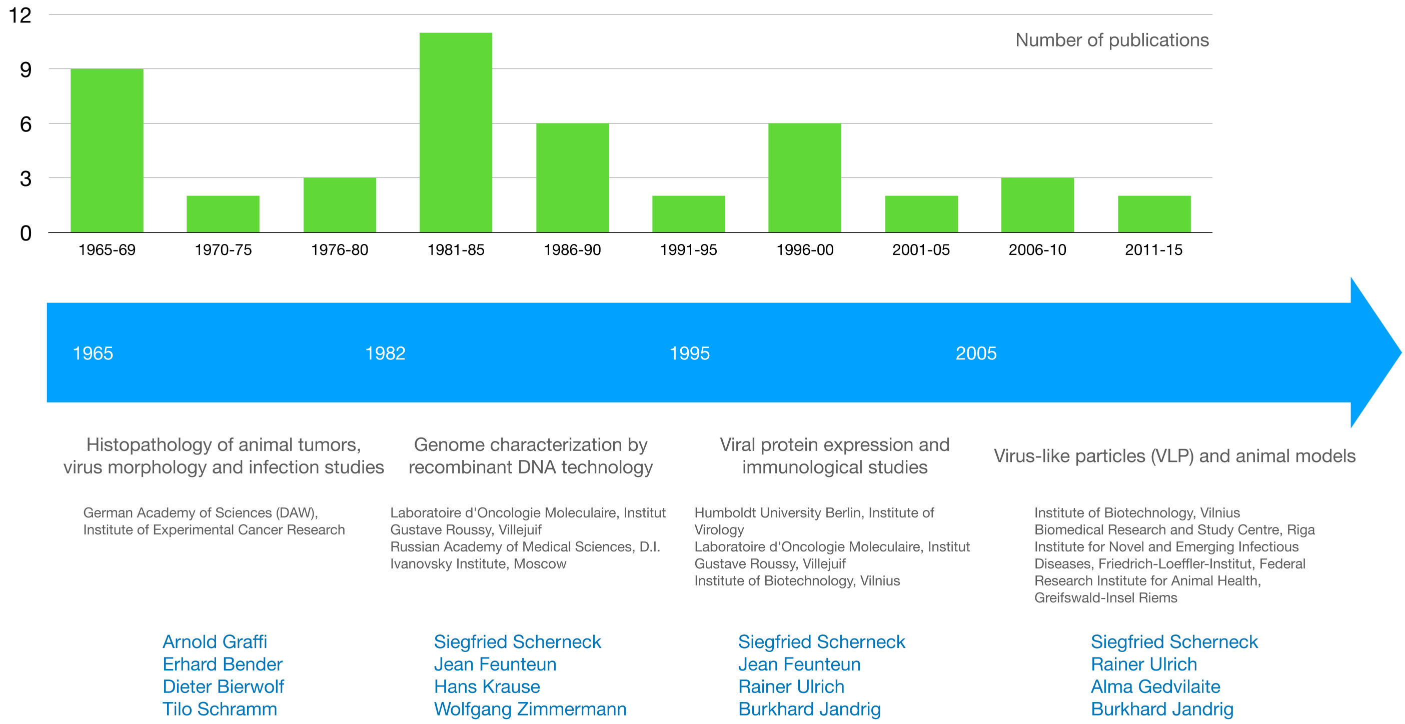

Figure S1. Time chart of objectives of hamster polyomavirus studies, involved main persons and number of publications when searching PubMed.

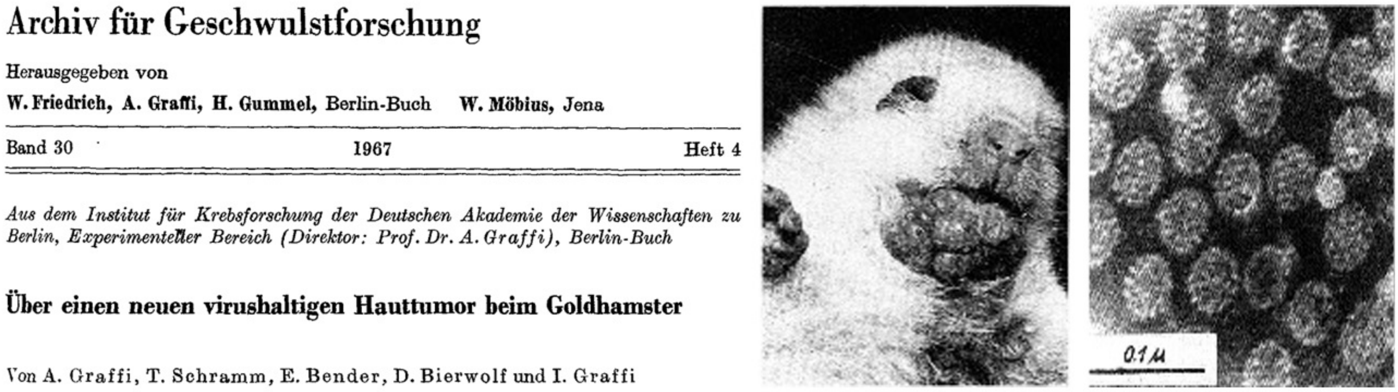
Archiv für Geschwulstforschung

Herausgegeben von

W. Friedrich, A. Graffi, H. Gummel, Berlin-Buch W. Möbius, Jena

Band 30 1967 Heft 4

Aus dem Institut für Krebsforschung der Deutschen Akademie der Wissenschaften zu Berlin, Experimenteller Bereich (Direktor: Prof. Dr. A. Graffi), Berlin-Buch

Über einen neuen virushaltigen Hauttumor beim Goldhamster

Von A. Graffi, T. Schramm, E. Bender, D. Bierwolf und I. Graffi

(a) **(b)** **(c)**

Figure S2. Screenshot of the title of the first paper describing a hamster polyomavirus **(a)**, typical multiple skin tumors **(b)** and a picture by electron microscopy **(c)** (x 260,000) [13]. The Journal was discontinued in 1991.
